# Importance of Neuropathological Diagnosis of Dementia Patients in Family Practice

**DOI:** 10.31662/jmaj.2018-0060

**Published:** 2019-06-06

**Authors:** Yukiko Tanaka, Masaki Ikeda, Ban Mihara, Yoshio Ikeda, Katsuya Sato, Tetsuyuki Kitamoto, Masaki Takao

**Affiliations:** 1 Department of Internal Medicine, Medical Corporation Taiseikai, Uchida Hospital, Gunma, Japan; 2 Department of Neurology, Gunma University Graduate School of Medicine, Gunma, Japan; 3 Mihara Memorial Hospital, Gunma, Japan; 4 Department of Locomotive Rehabilitation Science, Nagasaki University Graduate School of Biomedical Sciences, Nagasaki, Japan; 5 Department of Neurological Sciences, Tohoku University, Graduate School of Medicine, Miyagi, Japan; 6 Department of Neurology, Saitama Medical University International Medical Center, Saitama, Japan

**Keywords:** Creutzfeldt-Jacob disease, MM2-cortical type, MM1, prion, neuropathology, early-onset dementia, Alzheimer’s disease

## Abstract

**Introduction:**

Creutzfeldt–Jakob disease (CJD) is an important dementia disorder. However, clinical diagnosis can be difficult and delayed for many primary physicians caring for dementia patients. The aim of the present study was to describe clinical and neuropathological results of an individual with CJD who was seen by a community hospital. Our report may inform many primary physicians on understanding the significance of CJD.

**Methods:**

Clinical information was obtained from medical records. Neuropathological and biochemical analyses were performed using autopsied brain.

**Results:**

A 58-year-old Japanese man who had worked as a carpenter developed memory and executive function impairments. He was initially diagnosed as having Alzheimer’s disease based on clinical and neuroradiological analyses. Myoclonus was observed in the later stage of clinical course. Hyperintense lesions on diffusion-weighted images were observed in the cerebral cortex in later stage. Analysis of cerebrospinal fluid showed increased levels of total tau and phospho-tau protein. However, 14-3-3 protein and amyloid β (1–42) were normal. Genetic analysis of the *PRNP* gene showed methionine homozygosity at codon 129 and glutamate homozygosity at codon 219. The results of neuropathological analysis were consistent with sporadic CJD (MM2 cortical type with some type 1 pattern of 3F4 immunoreactivity). Western blot analysis of the frontal and cerebellar cortex revealed a type 2 and type 1 pattern of proteinase K (PK)-resistant prion protein, respectively. No Alzheimer’s pathology was present.

**Conclusions:**

Our experience may help primary physicians to assess dementia patients. Since atypical forms of prion disease are now well-established, we need to consider prion disease in dementia patients. Clinical examination alone is not enough for dementia workup; thus, we must understand the importance of neuropathological study and encourage autopsy to reach a definite diagnosis of dementia.

## Introduction

Prion disease is defined by the conversion of prion protein (PrP) from a normal type (PrP^C^) to an abnormal, infectious type (PrP^Sc^), which accumulates in the central nervous system and impairs neurological function with a lethal outcome ^(1), (2), (3), (4)^. Creutzfeldt–Jakob disease (CJD), a human prion disease, is clinically characterized by rapid progressive dementia, and pathologically, by spongiform changes and PrP^Sc^ accumulation in the brain ^(1), (2), (3), (4)^. Neuropathological or biochemical analysis of the brain is necessary to reach a definite diagnosis of CJD. There are different forms of human prion disease with different clinical presentations from classical CJD ^(1), (5)^. In atypical forms of prion disease, the patients may be diagnosed with another type of dementia such as Alzheimer’s disease, dementia with Lewy bodies, or vascular dementia. Since the number of dementia patients is increasing, many physicians are likely to see dementia patients in clinical practice. However, physicians who are not familiar with dementia disorders may not intuitively consider the possibility of prion diseases as a differential diagnosis of dementia. Here, we report an individual with CJD, who was initially diagnosed with early-onset Alzheimer’s disease (EOAD). Consequently, we want to emphasize the importance of considering prion diseases, as well as neuropathological analysis, when assessing patients with dementia.

## Materials and Methods

### Clinical information

The patient was predominantly observed by one of the authors (Y.T.) at Uchida Hospital (Gunma, Japan). The authors carefully reviewed all medical records. Certain information was obtained from family members of the patient and information obtained from the family physician’s referral letter was also used.

### Neuroimaging

**Figure 1.**
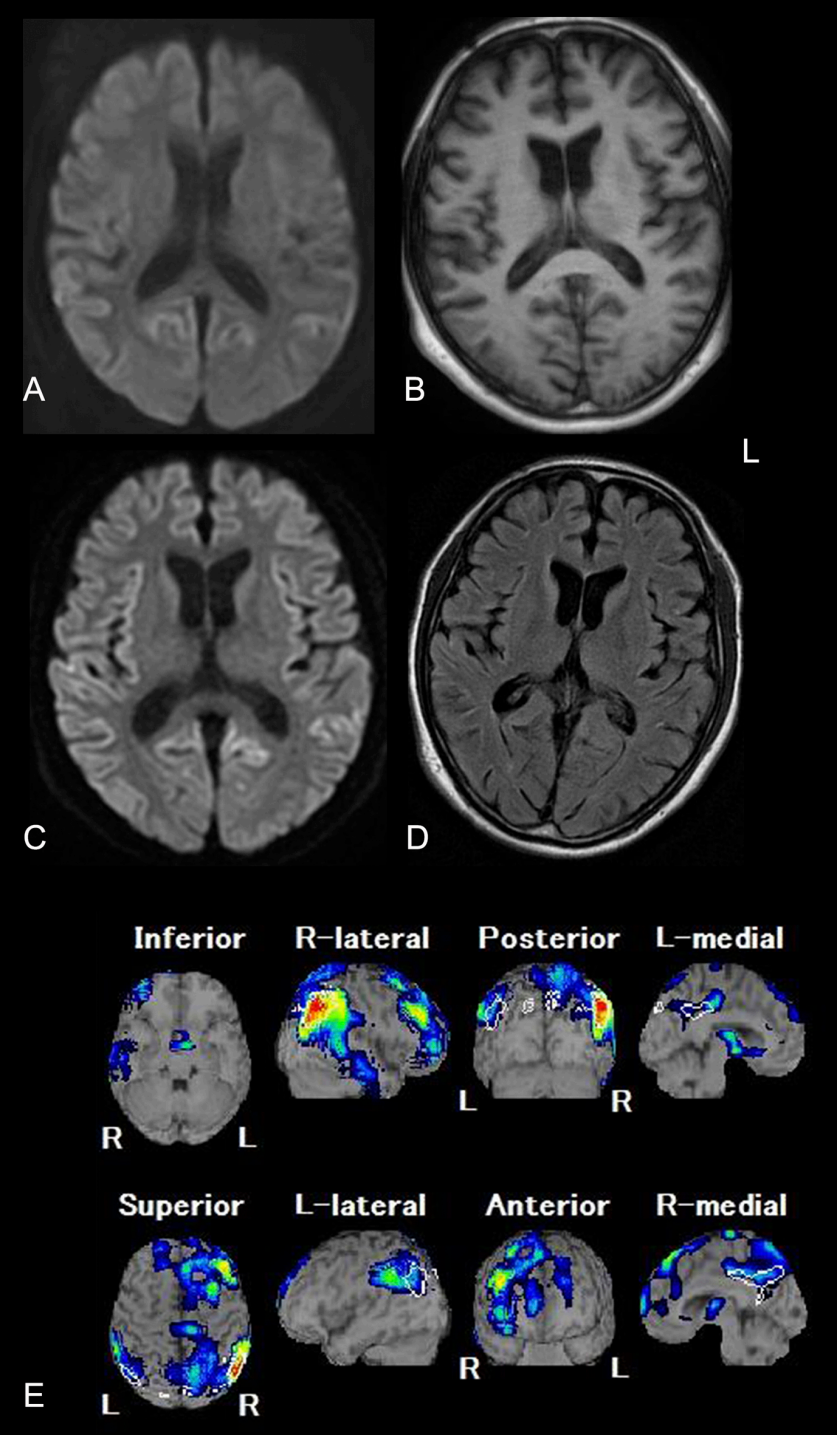
Neuroimaging of the present case. A. Diffusion weighted imaging (DWI) obtained on April 2013. Mild hyperintense lesions in the parietal and posterior lobes. B. T1-weighted image obtained on June 2013. Mild cerebral atrophy is present. DWI was not performed at the same time. C, D. Magnetic resonance imaging (MRI) obtained on December 2013. DWI (C) shows hyperintense lesions in cortical ribbons and caudate nuclei. No apparent signal abnormalities are present in fluid-attenuated inversion recovery (FLAIR) images (D). E. ^99m^Tc-ECD SPECT combined with the easy Z-score imaging system (eZIS) (July 2013) shows hypoperfusion at the level of the frontal, temporal, and parietal lobes, including the precuneus and posterior cingulate gyrus.

Repeated magnetic resonance imaging (MRI) scans were performed three times throughout the clinical course in several hospitals using different protocols. One technetium-ethyl cysteinate dimer single-photon emission computed tomography (^99m^Tc-ECD SPECT) scan was also carried out (Figure 1 A, B, C, D, E).

### Neuropathological examination

Autopsy was performed at the Mihara Memorial Hospital (Gunma, Japan). The right cerebrum, cerebellum, and brainstem were immediately frozen using dry ice and stored at −80℃ for future studies. The left hemisphere of the brain was fixed in 20% neutral buffered formalin (Wako, Osaka, Japan) for neuropathological analysis.

**Figure 2.**
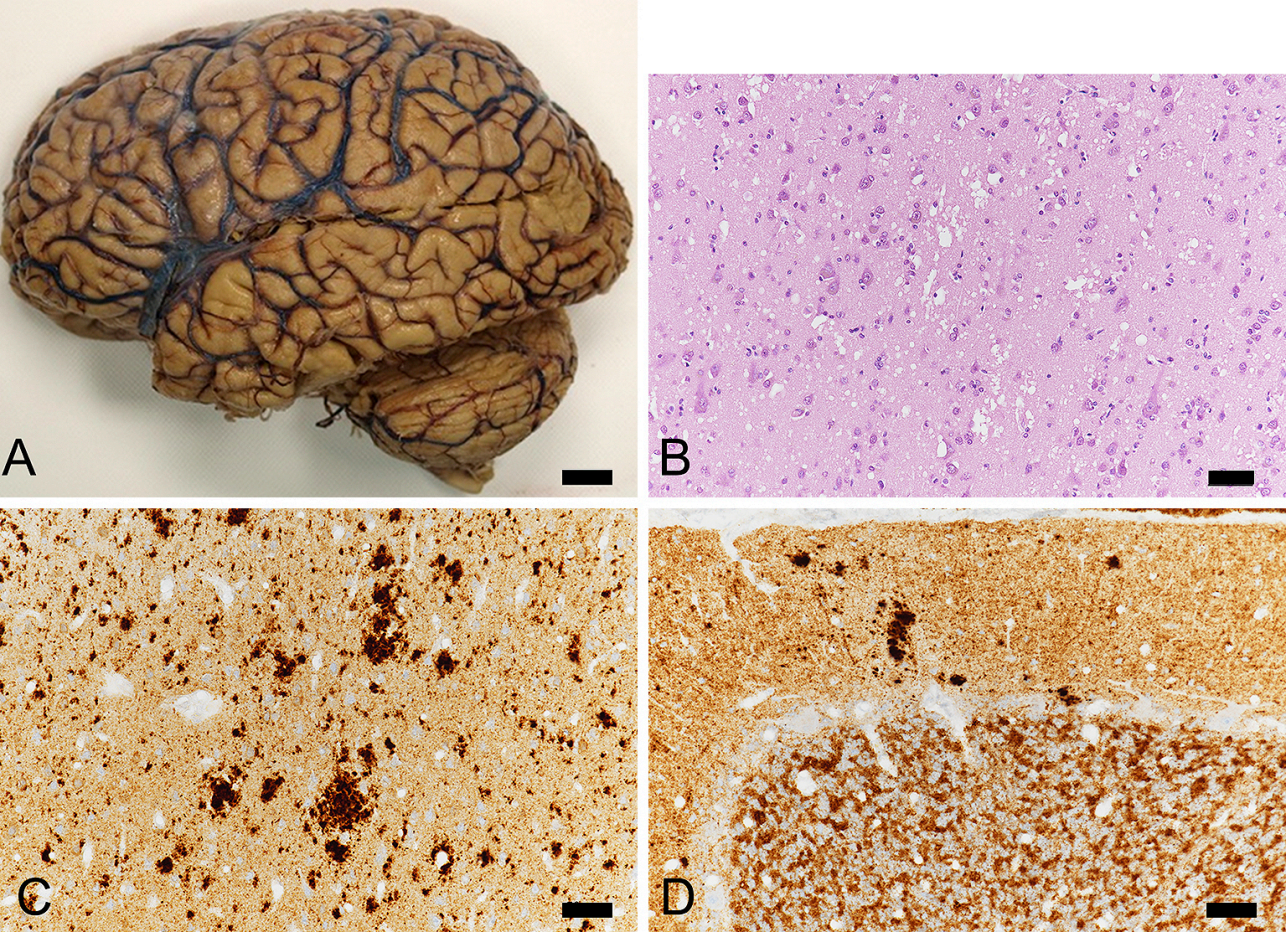
Neuropathological findings of the present case. A. Diffuse cerebral atrophy of the cerebral hemisphere after formalin fixation. Bar = 1cm. B. Large vacuolar changes are present in the temporal cortex. Hematoxylin and eosin staining. Bar =50μm. C. Coarse 3F4 immunoreactive deposits are seen in the temporal cortex. Immunohistochemistry using an antibody (3F4) raised against prion protein. Bar =50μm. D. Occasional dense 3F4 immunoreactive deposits in the cerebellar cortex are negative for thioflavin-S staining. Synaptic pattern 3F4 immunoreactivity was also present in the cerebellar cortex. Immunohistochemistry using an antibody (3F4) raised against prion protein. Bar =50μm.

The case was registered with our Brain Bank and neuropathologically analyzed according to the following protocols ^(6), (7)^. Samples were dissected from coronal slices of fixed brains from the following regions: superior and middle frontal gyri, anterior cingulate gyrus, superior and middle temporal gyri, motor and sensory cortices, insular cortex, calcarine cortex, amygdala, hippocampus, subiculum, parahippocampal gyrus, caudate nucleus, putamen, globus pallidus, thalamus, cerebellum, midbrain, pons, and medulla. Blocks were dehydrated in alcohol gradients, cleared in xylene, and embedded in paraffin. Brain tissue was cut into 6-µm thick sections. Sections were stained with hematoxylin and eosin (HE), Klüver–Barrera for myelin, and thioflavin staining. For immunohistochemical studies, the following specific monoclonal antibodies were used: amyloid β (Aβ) (11–28) (12B8, 1:100; IBL, Gunma, Japan), phospho-tau (AT8, 1:3,000; Innogenetics, Ghent, Belgium), phosphorylated α-synuclein (1:7,000, pSyn#64; Wako), phospho-TAR DNA-binding protein 43 (TDP-43) (s409/410, 1:7,000; COSMO BIO, Tokyo, Japan), and protease-resistant PrP (3F4: 1:200, Biolegend, USA, 1E4: 1:400, Cell Silences, USA). Sections were processed using a Ventana Discovery automated immunostainer (Roche, Basel, Switzerland). Sections were counterstained with hematoxylin (Figure 2).

### Western blot analysis of the brain tissue

Frozen frontal and cerebellar cortex were homogenized and western blot analysis of protease-resistant PrP was performed using monoclonal antibody 3F4, as well as type 1 and type 2 PrP^Sc^ specific antibodies according to a previous method ^(8)^.

### Approval code issued by the institutional review board (IRB) and the name of the institution(s) that granted the approval

All procedures performed in studies involving human participants were in accordance with the ethical standards of the institutional and/or national research committee, and with the 1964 Helsinki declaration and its later amendments or comparable ethical standards. The clinical and neuropathologic studies of prion diseases and brain bank of Mihara Memorial Hospital were approved by the Institutional Review Board of Mihara Memorial Hospital (072-01, 084-02, 085-01).

## Results

### Clinical presentation

A 58-year-old Japanese man who had worked as a carpenter developed memory and executive function impairment. Apart from a mild depressive condition after divorcing his wife at age 52, he did not show any significant medical illness. In February 2013, his mother noticed that he occasionally forgot to bring his work tools to work and frequently left work early without a specific reason. His working ability became slower and less accurate. His cognitive performance deteriorated because of attention deficits and amnesia. He stopped drinking alcohol, yet lost his activities of daily living, and it finally became difficult for him to continue with work.

Two months after onset, he was seen by a family physician and diagnosed with EOAD. At that time, he had confused his son for a friend when the son had visited his home. He also had an episode where he drove his car with a flat tire for more than 100 km. We reviewed diffusion weighted imaging (DWI) of MRI performed at this time and identified possible hyperintense lesions of the cerebral cortex (Figure 1A). However, the findings were not considered significant at the time.

As his cognitive dysfunction rapidly declined, he was referred to our hospital (Uchida Hospital) for further evaluation in May 2013 and examined by one of the authors (Y.T.). Neuropsychological examination revealed severe impairment of cognitive status including: 17/30 on Mini-Mental State Examination (MMSE), 17/30 on Hasegawa Dementia Rating Scale-Revised (HDS-R), and 39.3 on Alzheimer’s Disease Assessment Scale-cognitive subscale (ADAS-Cog). The Geriatric Depression Scale (GDS) suggested no depressive state. Brain MRI (1.5T, T1-weighted images [T1WI]) showed atrophy of the frontal and temporal lobes in both cerebral hemispheres (Figure 1B). Unfortunately, DWI and T2-weighted images (T2WI) were not performed. Nonetheless, based on his rapid cognitive decline, CJD was suspected instead of EOAD. Thus, we performed an examination of cerebrospinal fluid (CSF) and found increased levels of total tau protein (2,188 pg/mL; cut off 1,300) and phospho-tau-181 (82.64 pg/ml; cut off 45). In contrast, 14-3-3 protein was measured at 180.4 µg/mL (<500) using semiquantitative assay. Furthermore, Aβ1–42 was not decreased (399.23 pg/ml: 113.82±48.84 for AD and 379.25±144.45 for non-demented subjects ^(9)^, Wako Pure Chemical Industries). Real-time Quaking-Induced Conversion (RT-QUIC) was not carried out. Genetic analysis of the *PRNP* gene showed methionine homozygosity at codon 129 and glutamine at codon 219 without any mutation. Routine blood examination showed no significant abnormalities.

For further evaluation, the neurologist at Gunma University Hospital also examined the patient. Accordingly, his MMSE was recorded as 11, frontal assessment battery (FAB) was 6, and GDS was 1. Neurological examination showed dementia and ideational apraxia, as well as truncal ataxia, incoordination of both lower limbs, and increased deep tendon reflexes of both lower limbs. Tremor and myoclonus were not observed. ^99m^Tc-ECD SPECT combined with the easy Z-score imaging system (eZIS) revealed decreased cerebral blood flow in the posterior cingulate gyrus, precuneus, and parietal to frontal lobes (Figure 1E). Based on imaging analyses, Alzheimer’s disease was suspected.

In September 2013, seven months after onset, he was again seen at our hospital (Uchida Hospital). He had developed irritability and aggressive behavior and refused medical care from his family members. He frequently fell because of truncal ataxia and was having increasing difficulties with walking. In December 2013, he was neither able to speak nor communicate. At this point, he showed occasional myoclonus. Repeated brain MRI showed high-intensity lesions in the cerebral cortex including the insular, frontal, temporal, and occipital lobes on DWI (Figure 1D, E). There were no distinct high-intense lesions in the basal ganglia and thalamus. We were finally convinced that the clinical and neuroradiological conditions were consistent with CJD.

The patient was scheduled for admission to a welfare facility for severely demented patients to support total activities of daily living. In February 2014, at age 59, he developed akinetic mutism without tremor or myoclonus. In the same month, he died of intestinal obstruction. After obtaining consent from his family members, the body was transferred to Mihara Memorial Hospital to perform an autopsy.

### Neuropathological analysis

The fresh brain weighed 1,350 grams. After formalin fixation, the brain showed mild to moderate atrophy (Figure 2A). There was moderate neuronal loss and gliosis, as well as severe spongiform changes in the cerebral cortex. Relatively large vacuoles that appeared to fuse with adjacent vacuoles were observed (Figure 2B). In the motor cortex and hippocampus, spongiform changes were relatively mild. The inferior olivary nucleus was well preserved. In the cerebellum, there were slight spongiform changes in the cortex, along with gliosis.

3F4 immunocytochemistry showed coarse and granular deposits in the cerebral cortex (Figure 2C) and basal ganglia. Those coarse deposits were also immunoreactive for 1E4 antibody. Synaptic patterns of 3F4 immunoreactive deposits were also present. In the cerebellar cortex, there were 3F4 immunoreactive synaptic patterns as well as dense deposits (Figure 2D), which were negative for thioflavin-S staining. These neuropathological findings are consistent with MM2 cortical type (MM2C) ^(1)^ with some type 1 pattern of PrP^Sc^ immunoreactivity.

Aβ immunoreactive deposits were mild (Thal phase 1 ^(10)^) and AT8 immunoreactive deposits sparsely seen in the parahippocampus. AT8 immunoreactive small neuritic profiles ^(11)^ were also observed. No α-synuclein immunoreactive deposits were present. Therefore, AD neuropathology was not observed. Moreover, there was no evidence of cerebrovascular disease, vascular brain injury, or hippocampal sclerosis.

### Western blot analysis

**Figure 3.**
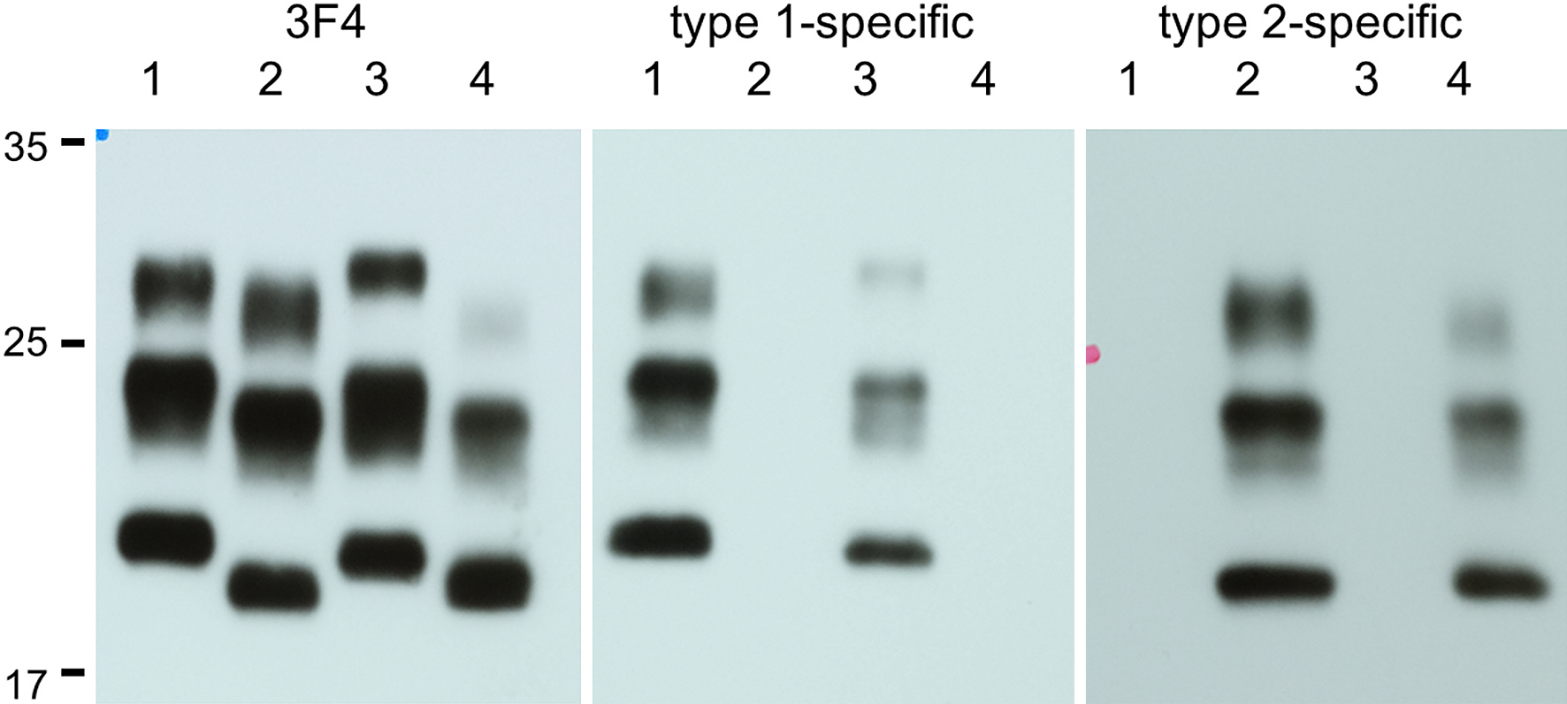
Western blot analysis of the patient. Western blot patterns of PrP^Sc^ from the frontal (Lane 2) and cerebellar (Lane 3) show type 2 and type 1, respectively. The results are also confirmed using specific antibodies for type 1 and type2 PrP. Lane 1: MM1 control, Lane 2: frontal cortex of the case, Lane 3: the cerebellar cortex from the case, Lane 4: MM2 control. Left side, molecular weight marker (kilodaltons)

Western blot analysis confirmed type 2 and type 1 PrP^Sc^ using frozen tissue of the right frontal and cerebellar cortex, respectively (Figure 3) ^(1), (12)^.

## Discussion

Based on the clinical course and autopsy findings, the present case was finally diagnosed with sporadic CJD (sCJD) of MM2C ^(1), (12)^. In addition, we confirmed that type 1 PrP^Sc^ accumulation in the cerebellum. The case was clinically characterized by relative rapid worsening of dementia and the deterioration of motor function, which led to death within one year. Thus, we want to emphasize the importance of neuropathological diagnosis of dementia patients before reaching a final diagnosis.

In Japan, the Prion Disease and Slow Virus Infection Surveillance Study Group confirmed 3,278 patients with prion disease in the period from 1999 to 2018 (http://prion.umin.jp/survey/survey.html accessed on November 10, 2018). The incidence rate was 1.4/1,000,000 persons, which is a two-fold increase from 0.7 in 1999. Mean age at onset was 68.8 years and mean duration of disease was 17.2 months (ranging from 2 to 117 months). Except for the genetic form of prion diseases, definite diagnosis of sporadic form of prion diseases was not confirmed unless neuropathological and biochemical analyses.

After referral to our hospital, CJD was clinically suspected in our case because of his rapid clinical deterioration compared with the classical course of AD. Based on diagnostic criteria of CJD, the present case was assigned a possible diagnosis of CJD. In fact, he showed high levels of total tau and phospho-tau protein with a normal range of Aβ1-42 in CSF. Accordingly, we were still considering the possibility that he had AD and not CJD due to negative 14-3-3 protein in CSF and abnormal pattern of SPECT imaging.

We did not carry out DWI and FLAIR brain images on his first visit to our hospital. Furthermore, the first DWI scan performed by general physician was considered normal (Figure 1A). Consequently, we may have missed the opportunity to detect a DWI abnormality ^(13)^ in the early course of the patient’s disease. The interpretation of MRI–DWI for individuals with CJD at early clinical course needs more analysis in the future. Since MRI images were carried out in different institutions and conditions, it was difficult to compare those images using standardized methodology. Electroencephalography was not available in our hospital and not performed at the University hospital. It is difficult to perform a complete evaluation for dementia patients in a primary clinical setting. Therefore, primary physicians must be aware of the importance of repeated MRI imaging, including DWI and FLAIR, for dementia patients until a final diagnosis is obtained.

Based on neuropathological and molecular studies, we classified the present case as MM2C of sCJD, with some type 1 pattern of 3F4 immunoreactivity. MM2C is a relatively rare condition and shows progressive dementia for several months as well as disturbances of higher cognitive functions, including aphasia and apraxia ^(1), (14)^. The progression of dementia is generally slower than classical CJD (MM1). As shown in the present case, myoclonus may be present in the later stage of clinical course. Thus, MM2C CJD patients may be misdiagnosed with another type of dementia.

Of particular interest, the present case showed cerebellar ataxia, some spongiform changes, and 3F4 immunoreactive deposits in the cerebellar cortex. Cerebellar pathology is not a common finding in sCJD of MM2C ^(15)^. Even in the case of MM1+2C, the cerebellar pathology is also relatively rare. In contrast, it is reported that MM2C+1 shows synaptic pattern of 3F4 immunoreactivity in the molecular layer of the cerebellum ^(12)^. Therefore, the present case may be classified to MM2C+1 ^(12)^. However, the present case also had synaptic pattern of 3F4 immunoreactivity in the cerebral cortex and coarse deposits in the cerebellar cortex. Further analysis would clarify the clinical and molecular correlation of prion diseases.

In conclusion, we report a case of sCJD MM2C with type 1 accumulation in the cerebellum that showed rapidly progressive dementia. Our experience is helpful for many primary physicians in assessing dementia patients. We must not rule out the prion diseases based on neuroimaging analysis. Needless to say, the careful and repeated evaluation of the dementia patient is important to reach the diagnosis of prion diseases. Since atypical forms of prion disease are now well-established, we must always consider prion disease as a differential diagnosis of dementia. In addition to appropriate laboratory examinations, it is important to understand that autopsy analysis is still only a methodology to reach a definite diagnosis of dementia. Our approach of collaborating family practice and neuropathological analysis facilitates the improvement of dementia care.

## Article Information

### Conflicts of Interest

None

### Sources of Funding

This work was supported in part by Research on Policy Planning and Evaluation for Rare and Intractable Diseases, Health and Labour Sciences Research Grants, The Ministry of Health, Labour and Welfare, Japan grant numbers 18058959, 17933542, and Japan Society for the Promotion of Science (JSPS) KAKENHI grant number JP18K06506 to Masaki Takao.

### Acknowledgement

We thank Mitsutoshi Tano and Shoken Aizawa for technical support. We thank Rachel James, Ph.D., from Edanz Group (www.edanzediting.com/ac) for editing a draft of this manuscript.

### Author Contributions

Yukiko Tanaka: design of the work, primary physician, drafting the work

Masaki Ikeda: clinical analyses

Ban Mihara: autopsy analyses

Yoshio Ikeda: clinical analyses

Katsuya Sato: analysis of cerebrospinal fluid

Tetsuyuki Kitamoto: analysis of molecular and biochemical analysis

Masaki Takao: neuropathologic analysis, revising manuscript critically for important intellectual content

### Approval by the Institutional Review Board (IRB)

All procedures performed in studies involving human participants were in accordance with the ethical standards of the institutional and/or national research committee, and with the 1964 Helsinki declaration and its later amendments or comparable ethical standards. The clinical and neuropathologic studies of prion diseases and brain bank of Mihara Memorial Hospital were approved by IRB of Mihara Memorial Hospital (072-01, 084-02, 085-01).
